# Gait variability is affected more by peripheral artery disease than by vascular occlusion

**DOI:** 10.1371/journal.pone.0241727

**Published:** 2021-03-31

**Authors:** Hafizur Rahman, Iraklis I. Pipinos, Jason M. Johanning, Sara A. Myers

**Affiliations:** 1 Department of Biomechanics, University of Nebraska at Omaha, Omaha, Nebraska, United States of America; 2 Department of Surgery, Veterans’ Affairs Medical Center of Nebraska and Western Iowa, Omaha, Nebraska, United States of America; 3 Department of Surgery, University of Nebraska Medical Center, Omaha, Nebraska, United States of America; Glasgow Caledonian University, UNITED KINGDOM

## Abstract

**Background:**

Patients with peripheral artery disease with intermittent claudication (PAD-IC) have altered gait variability from the first step they take, well before the onset of claudication pain. The mechanisms underlying these gait alterations are poorly understood.

**Aims:**

To determine the effect of reduced blood flow on gait variability by comparing healthy older controls and patients with PAD-IC. We also determined the diagnostic value of gait variability parameters to identify the presence of PAD.

**Methods:**

A cross-sectional cohort design was used. Thirty healthy older controls and thirty patients with PAD-IC walked on a treadmill at their self-selected speed in pain free walking (normal walking for healthy older controls; prior to claudication onset for PAD) and reduced blood flow (post vascular occlusion with thigh tourniquet for healthy older controls; pain for PAD) conditions. Gait variability was assessed using the largest Lyapunov exponent, approximate entropy, standard deviation, and coefficient of variation of ankle, knee, and hip joints range of motion. Receiver operating characteristics curve analyses of the pain free walking condition were performed to determine the optimal cut-off values for separating individuals with PAD-IC from those without PAD-IC.

**Results and discussion:**

Patients with PAD-IC have increased amount of variability for knee and hip ranges of motion compared with the healthy older control group. Regarding the main effect of condition, reduced blood flow demonstrated increased amount of variability compared with pain free walking. Significant interactions between group and condition at the ankle show increased values for temporal structure of variability, but a similar amount of variability in the reduced blood flow condition. This demonstrates subtle interactions in the movement patterns remain distinct between PAD-IC versus healthy older controls during the reduced blood flow condition. A combination of gait variability parameters correctly identifies PAD-IC disease 70% of the time or more.

**Conclusions:**

Gait variability is affected both by PAD and by the mechanical induction of reduced blood flow. Gait variability parameters have potential diagnostic ability, as some measures had 90.0% probability of correctly identifying patients with PAD-IC.

## 1. Introduction

Peripheral artery disease (PAD) is a common cardiovascular disorder that is associated with poor health outcomes, immobility, and physical dependence [1–3]. The most common symptom is intermittent claudication, a cramping pain that occurs in the calves, thighs and/or buttocks brought on by physical activity and relieved with rest. Patients with peripheral artery disease who experience intermittent claudication (PAD-IC) are also significantly compromised by decreased physical activity and a higher prevalence of falls [4–6]. Advanced biomechanical evaluations have been undertaken to elucidate the mechanisms responsible for these problems [7–12]. The results of these works demonstrate that the gait changes are driven by pathophysiological alterations that occur long before intermittent claudication symptoms drive patients to see a physician for pain during walking [6, 13, 14].

The older population is at higher risk for developing PAD, as statistics show that twenty-nine percent of individuals over the age of 70 years are affected by PAD [15]. Although patients with PAD-IC experience similar types of functional problems as older individuals (decreased push-off kinetics and higher rates of falling due to the decreased physical ability and strength), gait alterations are more severe in patients with PAD-IC [4, 5, 9, 16]. Risk factors for falling in older individuals are similar as those for patients with PAD-IC, including gait and balance impairments [17]. The incidence of PAD in older individuals makes it important to dissect the different pathophysiologic mechanisms that contribute to the falls and mobility problems in older adults with PAD compared with the mechanisms operating in the normal aging process. Further, a minimally invasive method that allows the detailed measurement of the severity of functional compromise in PAD and the monitoring of changes in the disease progression would also be a useful clinical tool.

Gait variability refers to the natural alterations that occur within and across gait cycles. The measurement of gait variability is a method that has shown promise in predicting falls, distinguishing between pathological and healthy individuals, and as an indicator of overall health of the biological system [18, 19]. Traditionally, movement variability was thought of as “noise”, but the current consensus is that it is inherent within all biological systems [20, 21]. The idea of “healthy variability” was originally put into application in the study of heart rhythm, but most recently has been studied in the context of gait. Gait patterns exhibit a similar pattern of ordered but varying pattern that can be described with mathematical scaling techniques and fractal analysis [22–24]. It is thought that the ordered variations in the steady state output of healthy systems represent the underlying physiologic capability to perform flexible adaptations to everyday and second by second stresses placed on the human body [25–27]. Consequently, the normal variability of human gait is the product of continual adjustments to the always changing environmental and morphological conditions affecting normal walking.

Alterations in gait variability have been found in numerous pathological populations, with some exhibiting deviations towards less order and others more order in gait [28–34]. Previous studies have identified that patients with PAD-IC have a significant increase in gait variability as compared with healthy older controls, indicating a less ordered pattern and significant deterioration of the locomotor system [7, 8]. The specific mechanisms leading to gait variability alterations from the “optimal” levels have not been elucidated. The muscle discomfort of intermittent claudication is commonly thought to be the factor that is responsible for the mobility problems in patients with PAD-IC [35], but in both gait biomechanics and gait variability studies, the patients have significantly altered gait from the first step they take and well before pain onset [7, 8]. Previous work from our and other groups points to abnormal lower extremity blood flow and underlying cellular abnormalities in the lower extremity muscles and nerves as possible mechanisms contributing to differences in the gait biomechanics and variability patterns as compared to healthy older controls [36–45].

Previous work from our group determined the role of walking-induced ischemia and its related muscle pain symptoms in the alterations of gait variability in patients with PAD-IC [46, 47]. We did this by examining healthy younger and healthy older controls before and after an induced vascular occlusion using a tourniquet placed at the level of the subject’s thigh [46, 47]. Both studies indicated gait variability was significantly altered during the reduced blood flow condition. However, the magnitude of changes during reduced blood flow were less than the magnitude of gait variability alterations seen in patients with PAD-IC. The average increases in gait variability from pain free to reduced blood flow conditions for the healthy younger and healthy older controls were 21% and 11.9%, respectively [46, 47]. In comparison, patients with PAD-IC have gait variability values that are 48% higher than healthy younger and 34% higher than healthy older controls [8].

The current study builds on our previous work. The primary aim of this study was to compare the gait variability between healthy older controls and patients with PAD-IC during pain free and reduced blood flow walking conditions. We hypothesized that gait variability values would be higher in patients with PAD-IC compared to healthy older controls during both pain free and reduced blood flow walking conditions. We also hypothesized that gait variability values would be increased during the reduced blood flow condition as compared to the pain free walking condition for both healthy older controls and patients with PAD-IC.

The current screening method for PAD is through measurement of ankle-brachial index in a vascular laboratory. This test is considered a specialized test that requires a separate medical appointment and it is only moderately correlated with functional outcomes [48]. Gait variability may provide an early indicator of functional problems, assess improvements following treatments, or provide an alert for declines that can occur with disease progression. Eventually, wearable devices may make it possible to capture gait variability in a clinical or home setting. For gait variability to be useful, we must first establish threshold gait variability values which indicate the presence of PAD. Therefore, a secondary aim was to test gait variability as a discriminant tool using a receiver operating characteristics curve. We hypothesized gait variability would provide acceptable discrimination between individuals with PAD-IC and those without PAD-IC.

## 2. Materials and methods

### 2.1 Participants

A cross-sectional cohort design was used for this research study. The study was approved by the Institutional Review Board at the Nebraska-Western Iowa Veteran Affairs Medical Center and University of Nebraska Medical Center. PAD can occur in adults of any age, but the prevalence of the disease starts to increase around the age of 40 years [49, 50]. Therefore, healthy older controls and patients with PAD-IC ranging between 40–80 years old were only recruited for this study. Assuming a 0.01 level two-sided test, 30 healthy older controls and 30 patients with PAD-IC would give about 80% power to detect an effect size of 0.7 (medium to large effect by Cohen [51]) for the mean change in gait parameters. Thirty healthy older control participants (age: 60.1 ± 8.03 years, age range: 45–74 years, mass: 86.6 ± 16.1 kg, height: 176.7 ± 8.3 cm, gender: 25 males, 5 females) and thirty age-matched, symptomatic patients with PAD-IC with moderate bilateral arterial occlusive disease and bilateral claudication (age: 63.8 ± 9.10 years, age range: 48–80 years, mass: 81.1 ± 14.7 kg, height: 171.8 ± 5.1 cm, gender: 28 males, 2 females, ankle brachial index for right leg: 0.57 ± 0.21, ankle brachial index for left leg: 0.55 ± 0.25) participated in the study. Subjects typically experienced intermittent claudication in the calf, thigh, and/or buttocks. Prior to data collection, informed consent was obtained from all participants according to the guidelines of the Institutional Review Boards.

Patients were screened and evaluated by one of two board-certified vascular surgeons. The evaluation included a detailed history and physical examination. Patients with PAD-IC were excluded if they experienced rest pain, or pain or appreciable limitation during walking for any reason other than claudication [50]. Such significant limitations included inability to walk without an aid, cardiac, pulmonary, neuromuscular, or musculoskeletal or joint disease. Shoe inserts and footwear were worn as prescribed to the subjects. Healthy older control subjects had an ankle-brachial index ≥ 1.0 and no subjective or objective ambulatory dysfunction. Healthy older controls were excluded if they experienced any discomfort or limitation during walking.

### 2.2 Experimental procedures and data collection

The experimental tests were conducted at the Biomechanics Research Building at the University of Nebraska at Omaha. Upon arrival to the laboratory, subjects height, body mass, and anthropometric measures were taken. Healthy older controls had lower extremity blood flow measured by taking the systolic pressures at the brachial artery in the arm and the dorsal pedis and posterior tibial arteries at the ankle to confirm acceptable ankle-brachial index values. Patients with PAD-IC had ankle-brachial index values assessed in the same manner, however these measures were taken at the respective clinical site and did not need to be repeated in the biomechanics laboratory. Reflective markers were placed on specific anatomical locations of each subject’s lower limb using the systems of Vaughan et al. [52] and Nigg et al. [53] as previously described [8]. Subjects were then allowed to get familiar with walking on the treadmill (BodyGuard Fitness, St. Georges, QC, Canada). During familiarization, subjects were asked to select a comfortable walking speed, which was then identified as the self-selected walking speed and was used for all further testing.

An eight camera motion analysis system (Eagle cameras, Motion Analysis Corp, Santa Rosa, CA) was used to capture kinematics while subjects walked on the treadmill. Three-dimensional movements were acquired at 60 Hz using EVART software (Motion Analysis Corp, Santa Rosa, CA). The frequency of normal gait has an upper limit between 4 Hz to 6 Hz [54]. According to the Nyquist sampling theorem that suggests that the sampling rate must be at least twice as the highest frequency of the signal [55], 60 Hz was chosen as sampling frequency. Prior to recording the kinematics data, subjects stood in a calibration device to collect an anatomical reference position of each leg. This position was used to orient the lower extremity segments and served as the reference point for relevant joint angles. Data collection continued as follows for the healthy older controls and for patients with PAD-IC:

#### 2.2.1 Healthy older controls

Subjects walked on the treadmill at their self-selected pace for three minutes, which was considered as the pain free walking condition. Next, vascular occlusion was induced by placing thigh cuffs (Omron® Exactus Aneroid Sphygmomanometer Model 108MLNL, Kyoto, Japan) bilaterally on the upper thighs and occlusion tourniquets (CyberTech™ Mechanical Advantage Tourniquet MAT01, Maharashtra, India) just above the knee while subjects stood on the treadmill. The cuffs were inflated to 200 mm Hg and maintained for three minutes. The chosen level of pressure and time of occlusion is standard and has been used in previously published similar studies to induce ischemia in the legs [56–61]. After three minutes of occlusion, the thigh cuffs were removed, and the subjects immediately began walking on the treadmill. Three minutes of treadmill walking was recorded for post vascular occlusion and defined as the reduced blood flow condition for healthy older controls.

#### 2.2.2 Patients with PAD-IC

Patients walked on a treadmill at self-selected speed for three minutes or until the onset of claudication pain, whichever came first. This was the pain free walking condition. Subjects were then required to rest for a minimum of 10 minutes to ensure that they were relieved from claudication pain. Next, the treadmill was inclined to 10% grade and the subjects walked until the onset of claudication pain. Once pain was present, the treadmill was lowered to 0% grade and the patients walked on the level treadmill with claudication pain for as long as tolerable up to three minutes. This was defined as the reduced blood flow condition for patients with PAD-IC.

### 2.3 Data analysis

Coordinate trajectory data of each marker were exported and processed using custom codes in MATLAB software (MathWorks Inc, Natick, Mass). The data were analyzed unfilter to obtain the most accurate representation of the gait variability. Custom MATLAB software was used to calculate relative joint angle time series from the kinematic data for the ankle, knee, and hip for all trials as previously described [10, 47]. Joint kinematic variability has been shown to be a more sensitive measure of differences between groups than variability of stride characteristics (stride time, step time) [62]. All trials were cropped to 3300 data points, which is long enough to allow 30 continuous footfalls and is considered adequate for nonlinear analysis [23, 63]. For healthy older controls, the acceleration phase (treadmill getting up to speed) was not included into the analysis. For patients with PAD-IC, recording started when the treadmill reached 0% incline. We assessed gait variability using both linear and nonlinear analysis.

#### 2.3.1 Gait variability: Linear analysis

The linear analysis gives information regarding the amount of variability present in gait patterns and is used in conjunction with the nonlinear analysis [23]. Ranges of motion of the ankle, knee, and hip joint angles were calculated for the gait cycles in every trial. Peak discrete points (maximum and minimum in each cycle) were used as the gait events to calculate the ranges of motion by MATLAB codes as previously described [7, 8, 46, 47]. Means, standard deviations, and coefficients of variation ((standard deviation / mean) * 100) were then calculated for each variable. These calculations were made using custom written laboratory codes in MATLAB software (MathWorks Inc, Natick, MA, USA).

#### 2.3.2 Gait variability: Nonlinear analysis

The largest Lyapunov exponent and approximate entropy were utilized for this study. Linear analysis considers a few specific points in the series, but both the largest Lyapunov exponent and approximate entropy investigate the temporal structure of the entire time series of the joint angle. The largest Lyapunov exponent approximates the sensitivity of the locomotor system to perturbations by quantifying the exponential separation of nearby trajectories in the reconstructed state space of the joint angle time series. As nearby points of the state space separate, they diverge rapidly and can produce instability. The largest Lyapunov exponent ranges from zero in a stable system with little to no divergence (e.g., sine wave) to a larger value around 0.5 for an unstable system with a high amount of divergence (e.g., white noise) [23, 64, 65]. A deterministic or ordered signal that demonstrates mathematical chaos will have a largest Lyapunov exponent value between zero and 0.5 when it is calculated using the Wolf et al. algorithm implemented in the Chaos Data Analyzer [66]. To numerically calculate the largest Lyapunov exponent for each joint angle time series for each trial the *Chaos Data Analyzer* professional version (American Institute of Physics, Raleigh, NC, USA) was used [67]. The detailed description of actual calculation of the largest Lyapunov exponent was previously described [8, 46, 47]. No surrogation was performed in this experiment, as it has been performed for these groups previously [7, 8, 46, 47].

Approximate entropy was calculated to determine the repeatability present in each trial [23, 28, 68]. Approximate entropy gives information regarding the regularity of a time series by measuring the logarithmic probability that a series of data points at a certain distance apart will exhibit similar relative characteristics on the next incremental comparison with the state space [69–72]. Time series with a greater likelihood of remaining the same distance apart in the next incremental comparison with a state space (periodic and predictable) will result in approximate entropy values close to zero. Those data points that vary widely in distances between data points (irregular and not predictable) will result in high values close to two. For example, the approximate entropy value for a periodic time series like the sine wave will be close to zero and the value for a random time series like white noise will be close to two. A signal that demonstrates mathematical chaos will lie somewhere between zero and two. A more detailed description of the calculation of the approximate entropy was previously described [8, 46, 47].

#### 2.3.3 Receiver Operating Characteristics (ROC) curve analysis

Mean values for linear and nonlinear gait variability parameters from the pain free walking condition for both healthy older controls and patients with PAD-IC were utilized to construct receiver operating characteristics curves. First, logistic regression was performed independently for each gait variability dependent variable to determine if each variable was significantly associated with disease status (presence or absence of PAD). Next, the ROC curves were calculated for all variables with a significant relationship with disease status. The area under the curve, which gives the variable’s overall ability to predict the presence of PAD was calculated. Area under the curve values range from 0.0, which represents no ability to discriminate PAD from healthy older controls, to 1.0, which would give perfect discrimination of those with PAD from those without PAD [73–75]. The level of acceptable discrimination was set at 0.75 (75% of the area under the curve) for each variable [73, 75]. The area under the curve ranging from 0.70 to 0.79, 0.80 to 0.89, and 0.90 to 0.99 were interpreted as “acceptable”, “excellent”, and “outstanding” discrimination, respectively [73]. Prediction equations were calculated for all dependent variables that had area under the curve values greater than or equal to 0.75. Optimal cut-off points, percentage correct, *p* values, 95% confidence interval, sensitivity, and specificity values were calculated to provide information regarding the accuracy of each prediction equation. The probability of the presence of PAD for an individual observation (π(x)) is calculated from each prediction equation using the form of Eq (1), which is derived from the *logit* transformation. If π(x) is greater than the optimal cut-off point for the dependent variable, then the observation is classified as PAD. If π(x) is less than the cut-off point, the observation is classified as healthy.

$$\pi \left(x\right)=e^{\alpha +\beta x}/(l+e^{\alpha +\beta x})$$ (1)

### 2.4 Statistical analysis

Group and condition means for the standard deviation and the coefficient of variation of the range of motion, largest Lyapunov exponent values, and approximate entropy values were calculated for the ankle, knee, and hip joint angles. Data were normally distributed as checked by the Shapiro-Wilk test. For each dependent variable, a two by two mixed ANOVA was used to detect differences for the main effects of group (healthy older controls versus patients with PAD-IC) and condition (pain free versus reduced blood flow) factors. When a significant interaction was identified, Tukey tests were used for post-hoc analysis to identify significant differences between the group/condition combinations [76]. Statistical comparisons were performed using SPSS software (version 26, IBM, Armonk, NY). The level of significance was set at 0.05.

## 3. Results

### 3.1 Linear measures of variability

**Fig 1:**
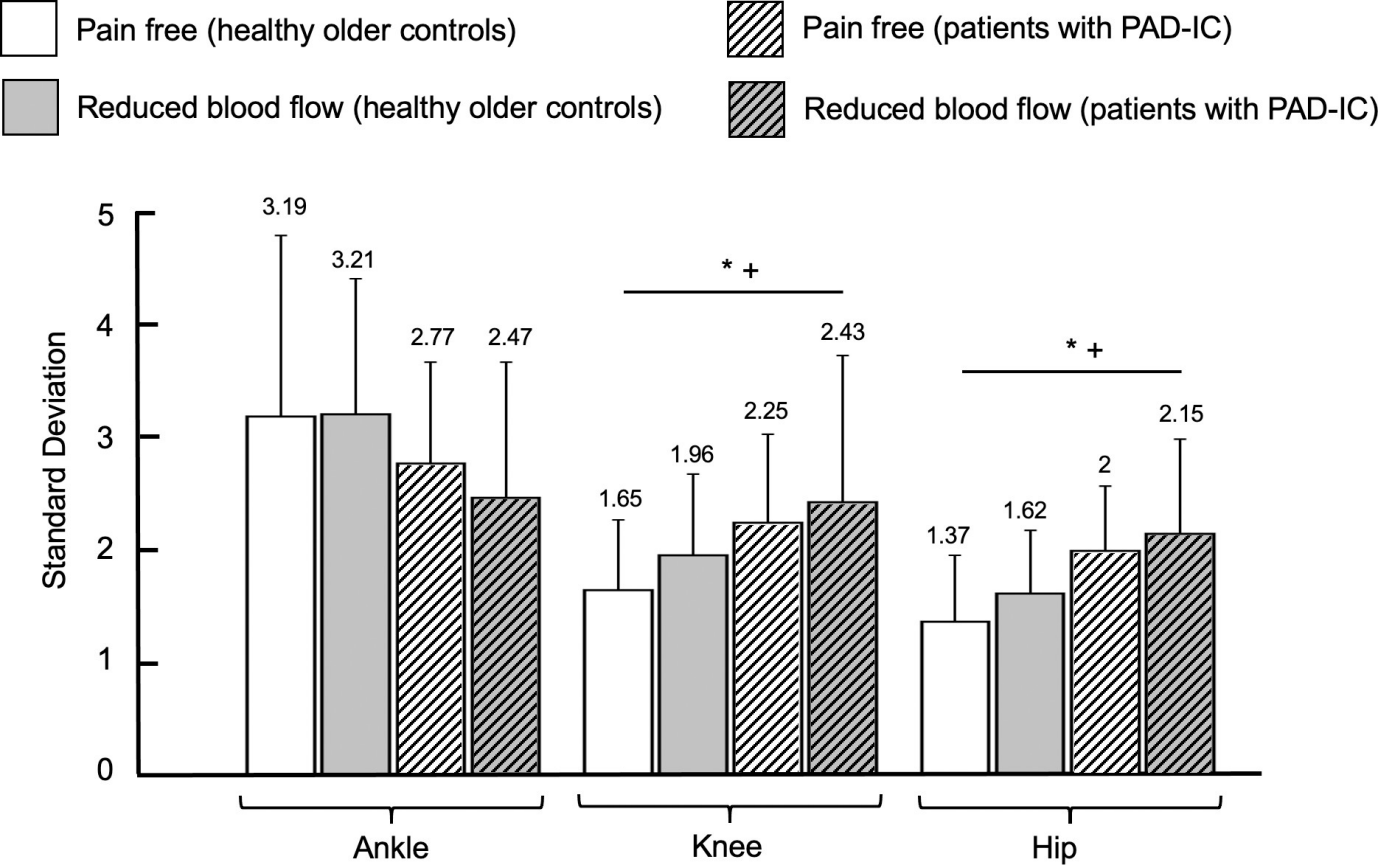
Standard deviation for healthy older controls and for patients with Peripheral Artery Disease with Intermittent Claudication (PAD-IC) in the pain free and reduced blood flow conditions for ankle, knee, and hip ranges of motion. * indicates significant differences between conditions (pain free vs. reduced blood flow, *p* < 0.05). + indicates significant differences between groups (healthy older controls vs. PAD-IC, *p* < 0.05). # indicates post-hoc tests where significant interaction exists between groups and conditions. Bar graphs represent the mean values and error bars represent the standard deviation. Mean value for each bar was mentioned at the top of the corresponding error bar.

**Fig 2:**
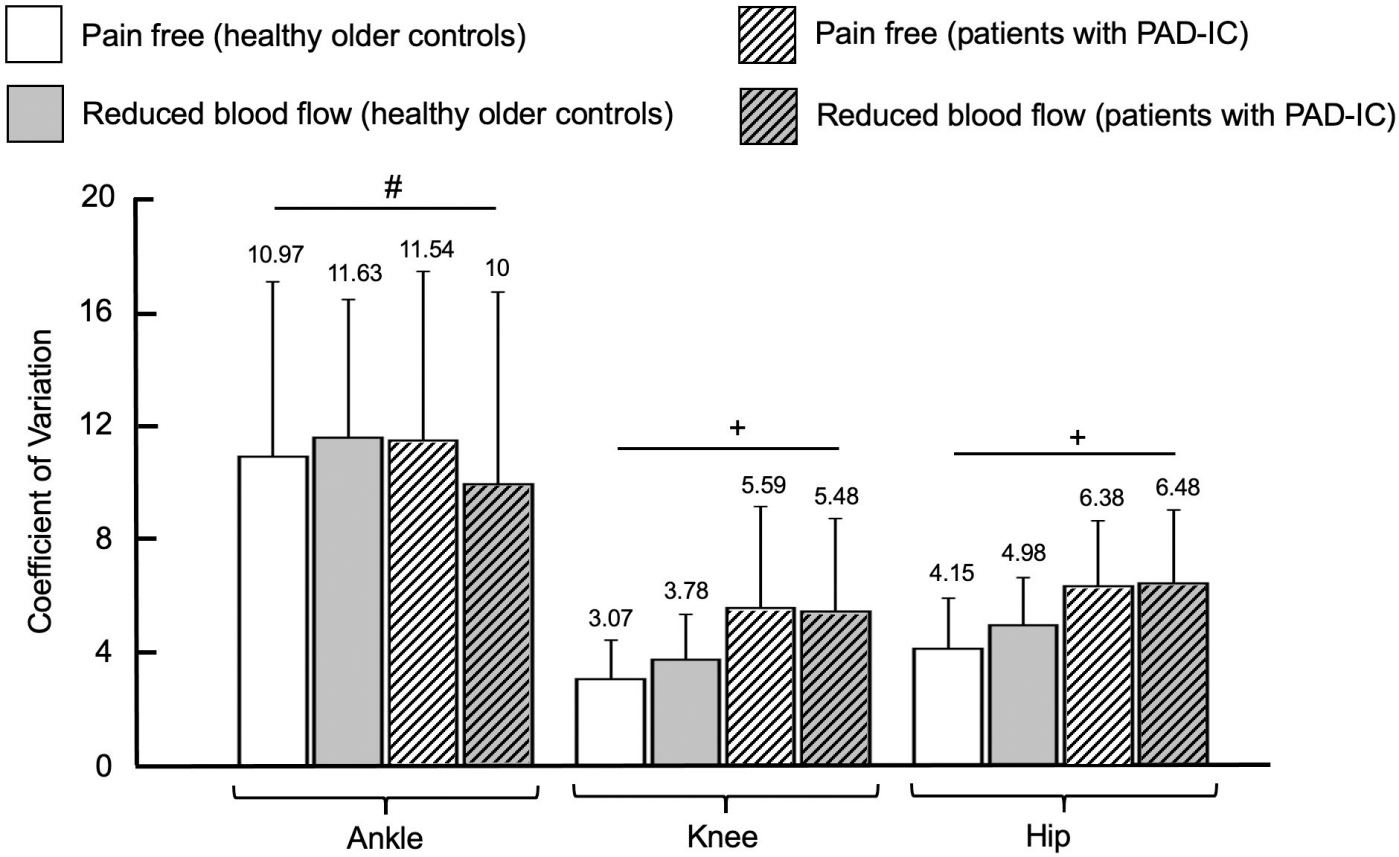
Coefficient of variation for healthy older controls and for patients with Peripheral Artery Disease with Intermittent Claudication (PAD-IC) in the pain free and reduced blood flow conditions for ankle, knee, and hip ranges of motion. * indicates significant differences between conditions (pain free vs. reduced blood flow, *p* < 0.05). + indicates significant differences between groups (healthy older controls vs PAD-IC, *p* < 0.05). # indicates post-hoc tests where significant interaction exists between groups and conditions. Bar graphs represent the mean values and error bars represent the standard deviation. Mean value for each bar was mentioned at the top of the corresponding error bar.

There were several differences between groups (healthy older controls versus patients with PAD-IC). Patients with PAD-IC exhibited increases in standard deviation and coefficient of variation values of the knee and hip ranges of motion as compared to healthy older controls (standard deviation: for knee, *p* = 0.01, and for hip, *p* < 0.001; coefficient of variation: for knee, *p* = 0.002, and for hip, *p* < 0.001; Figs 1 and 2). There were also significant differences for condition (pain free versus reduced blood flow). Specifically, the reduced blood flow condition had increases in standard deviation values of the knee and hip ranges of motion compared to the pain free walking condition (for knee, *p* = 0.02, and for hip, *p* = 0.03).

There was also a significant interaction between group and condition for the ankle range of motion coefficient of variation; patients with PAD-IC exhibiting increased values in the pain free condition as compared with the reduced blood flow condition (Fig 2). There were no other significant interactions between group and condition means. Collectively, the results of the linear analysis indicate that the PAD-IC group exhibited increased amount of variability for the knee and hip ranges of motion compared with the healthy older controls group. Additionally, the reduced blood flow condition demonstrated increased amount of variability for the knee and hip ranges of motion compared with the pain free walking condition.

### 3.2 Nonlinear measures of variability

**Fig 3:**
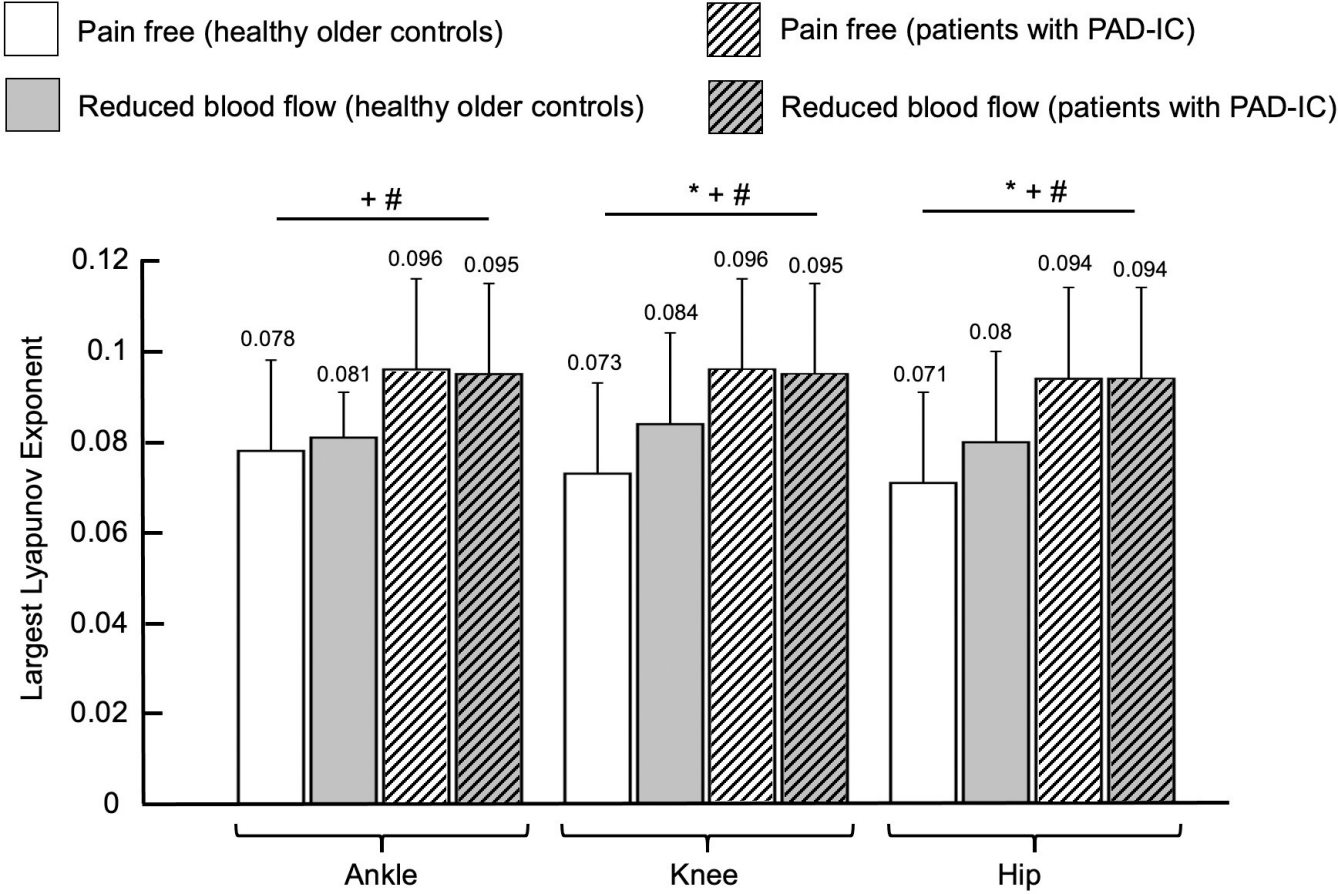
Largest Lyapunov exponent values for healthy older controls and for patients with Peripheral Artery Disease wth Intermittent Claudication (PAD-IC) in the pain free and reduced blood flow conditions for ankle, knee, and hip ranges of motion. * indicates significant differences between conditions (pain free vs. reduced blood flow, *p* < 0.05). + indicates significant differences between groups (healthy older controls vs. PAD-IC, *p* < 0.05). # indicates post-hoc tests where significant interaction exists between groups and conditions. Bar graphs represent the mean values and error bars represent the standard deviation. Mean value for each bar was mentioned at the top of the corresponding error bar.

**Fig 4:**
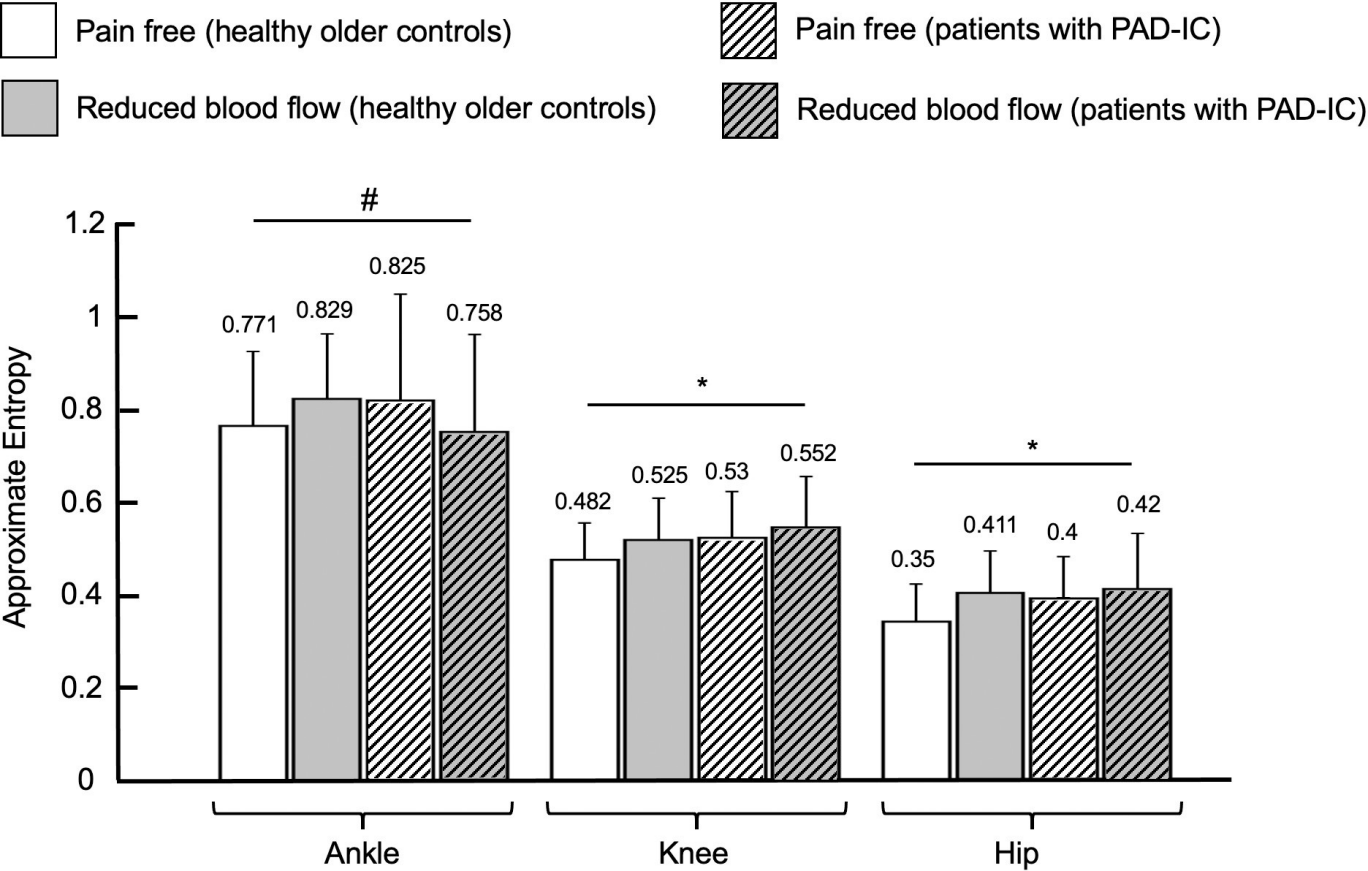
Approximate entropy values for healthy older controls and for patients with Peripheral Artery Disease with Intermittent Claudication (PAD-IC) in the pain free and reduced blood flow conditions for ankle, knee, and hip ranges of motion. * indicates significant differences between conditions (pain free vs. reduced blood flow, *p* < 0.05). + indicates significant differences between groups (healthy older controls vs PAD-IC, *p* < 0.05). # indicates post-hoc tests where significant interaction exists between groups and conditions. Bar graphs represent the mean values and error bars represent the standard deviation. Mean value for each bar was mentioned at the top of the corresponding error bar.

Regarding the effect of group, patients with PAD-IC showed increased values of the largest Lyapunov exponent for the ankle, knee, and the hip joint time series (for ankle, *p* < 0.001, for knee, *p* = 0.001, and for hip, *p* < 0.001; Fig 3), but there were no significant group differences for approximate entropy (Fig 4). For the effect of condition, the reduced blood flow condition exhibited increased values of the largest Lyapunov exponent and approximate entropy for both the knee and hip joint angle time series as compared to the pain free condition (largest Lyapunov exponent: for knee, *p* = 0.005, and for hip, *p* = 0.016; approximate entropy: for knee, *p* = 0.002, and for hip, *p* < 0.001; Figs 3 and 4).

There were significant interactions between group and condition for the ankle, knee, and the hip joint time series. At the ankle, the PAD-IC pain free and PAD-IC reduced blood flow conditions demonstrated significantly increased values for the largest Lyapunov exponent of the joint angle time series compared with the healthy older controls pain free and healthy older controls reduced blood flow values (Fig 3). Additionally, the approximate entropy of the ankle joint time series during the reduced blood flow condition of the healthy older controls was significantly increased compared to the pain free condition (Fig 4). At the knee, the largest Lyapunov exponent values of the joint angle time series were also increased in the PAD-IC pain free and the PAD-IC reduced blood flow conditions compared to both the healthy older controls pain free and healthy older controls reduced blood flow conditions (Fig 3). The reduced blood flow condition in the healthy older controls group was also significantly increased compared to the pain free condition for the largest Lyapunov exponent values of the knee joint time series. The significant interactions at the hip were identical to those at the knee, with the PAD-IC pain free and the PAD-IC reduced blood flow conditions having significantly increased largest Lyapunov exponent values compared to the healthy older controls pain free and healthy older controls reduced blood flow conditions, and the healthy older controls reduced blood flow being increased compared to the pain free condition (Fig 3).

Results of the nonlinear analysis demonstrate that overall, patients with PAD-IC have increased values of nonlinear analysis measures as compared to healthy older controls, regardless of the blood flow status. However, reduced blood flow also led to increases in values of measures of temporal structure of variability. The post hoc analyses further support these results as they generally show that patients with PAD-IC have higher values of nonlinear measures of variability during both conditions compared with both conditions in healthy older controls. These increased values indicate irregularity of the joint angle time series and increased divergence in consecutive strides of the joint movement patterns.

### 3.3 Receiver operating characteristics curve

**Fig 5:**
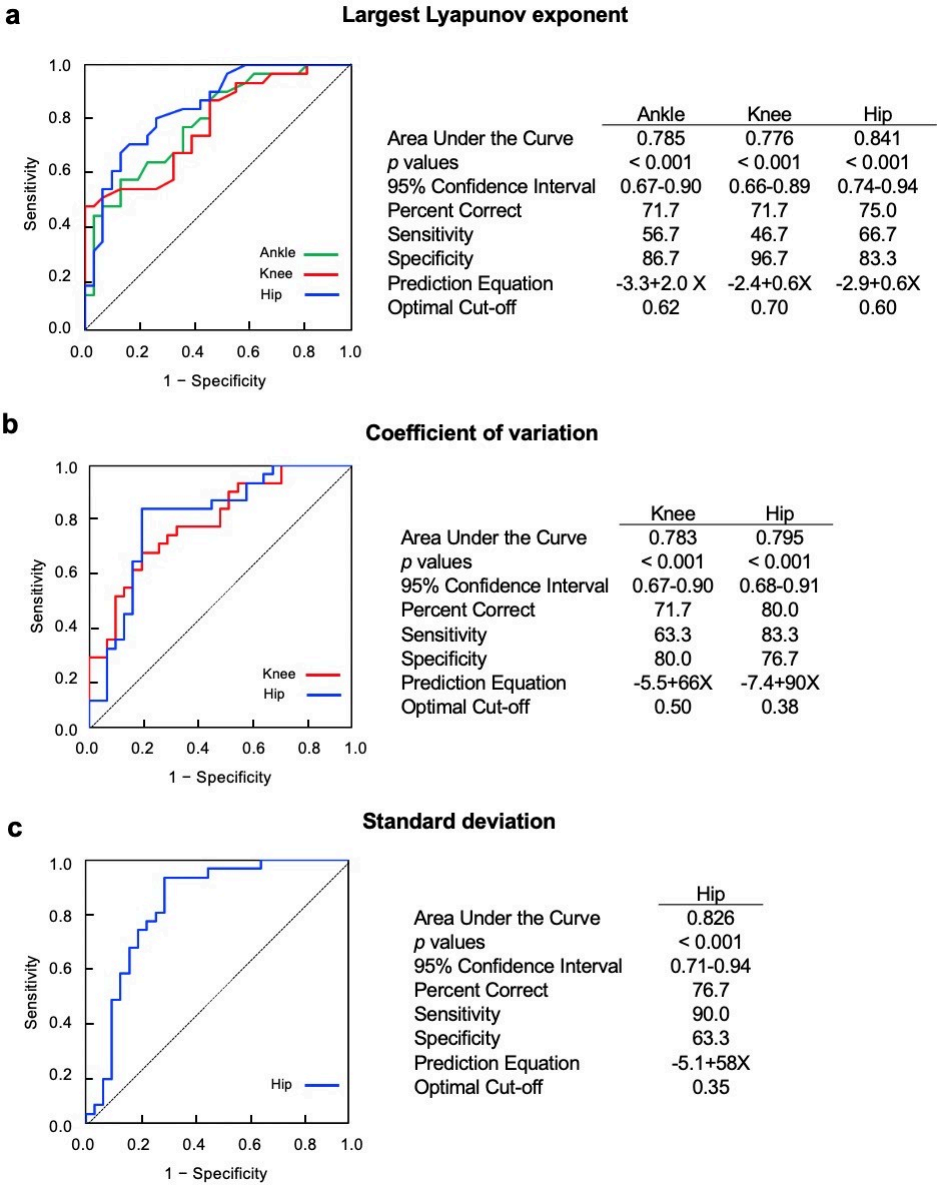
Results from Receiver Operating Characteristics (ROC) curve and logistic regression analyses. Six dependent variables have significant relationship exist with disease status and all of them have area under the curve ≥ 0.75. a) ROC curves for Largest Lyapunov exponent of ankle, knee, and hip ranges of motion, b) ROC curves for coefficient of variation of knee and hip ranges of motion, c) ROC curve for standard deviation of hip range of motion.

Of the twelve individual variables, six gait variability parameters had a significant relationship with disease status and also had area under the curve values ≥ 0.75. Those variables were standard deviation of the hip joint range of motion, coefficient of variation of the knee and the hip joint ranges of motion, and the largest Lyapunov exponent at the ankle, knee, and the hip joint angle time series (Fig 5). For these six variables, area under the curve values ranged from 0.776 to 0.841, which is interpreted as “acceptable discrimination” to “excellent discrimination” of the accuracy statistic. The percent of subjects correctly identified by individual variables ranged from 71.7% to 80.0%, while sensitivity ranged from 46.7% to 90.0% and specificity ranged from 63.3% to 96.7%. Optimal cut-off points and prediction equations are also reported in Fig 5. These results demonstrate that several gait variability variables provide acceptable to excellent discrimination between groups.

## 4. Discussion

This study sought to examine the effect of reduced blood flow as the central mechanism contributing to the walking limitations seen in PAD by evaluating lower extremity gait variability in healthy older controls and patients with PAD-IC during pain free walking and reduced blood flow walking conditions. Our first hypothesis was that gait variability would be different between healthy older controls and patients with PAD-IC. Results of our variability analyses supported this hypothesis, as variability values were significantly increased at the knee and hip ranges of motion in patients with PAD-IC. Temporal structure of variability values were also increased at all three joint angle time series for most measures when compared with healthy older controls. Our second hypothesis was that the reduced blood flow condition would demonstrate increased gait variability values compared with gait variability in the pain free walking condition. Regarding this hypothesis, our results were supported at the knee and hip joints for measures of amount and temporal structure of variability. However, the ankle data did not support this, as there were no significant differences for conditions for measures of amount nor temporal structure of variability. Interestingly, significant interactions between group and condition occurred at the ankle for largest Lyapunov exponent, approximate entropy, and coefficient of variation suggesting that the ankle is affected by more than just reduced blood flow. We also analyzed our data to see if the gait variability would be similar between the healthy older controls and the patients with PAD-IC in the reduced blood flow condition. We found that the two groups in the reduced blood flow condition had similar amount of variability but differed in the temporal structure of variability. The lack of significant interactions between the PAD-IC reduced blood flow and healthy older controls reduced blood flow conditions for the linear variability measures shows that the amount of variability is consistent between the conditions. However, the PAD-IC reduced blood flow condition had significantly increased values of the largest Lyapunov exponent for the ankle, knee, and hip joint angle time series and significantly decreased approximate entropy of the ankle joint time series as compared to the healthy older controls reduced blood flow condition. Thus, while the variability in the amount individuals move does not seem to change, the subtle interactions in the movement patterns remain distinct between the PAD-IC group and the healthy older controls in the reduced blood flow condition.

An additional objective of this study was to determine the sensitivity and specificity of gait variability parameters in predicting disease status (presence or absence of PAD) using a ROC curve analysis. We hypothesized that gait variability would be able to provide good discrimination between individuals with PAD-IC and those without PAD-IC. The ROC curve analysis of gait variability parameters supported this hypothesis. Specifically, a combination of six gait variability parameters could correctly identify disease status of subjects 70% of the time or more. These variables included the largest Lyapunov exponent at the ankle, knee, and the hip joint time series, the standard deviation of the hip range of motion, and the coefficient of variation of the knee and the hip joint ranges of motion. Area under the curve values for these parameters indicated acceptable to excellent discrimination between groups. The majority of PAD patients are asymptomatic especially at the early stages of the disease [37], therefore, a combination of gait variability parameters could be used as a diagnostic tool for identifying patients in the community that demonstrate gait characteristics that are abnormal and suggest the development of PAD.

The results of our current study suggest that gait variability is affected both by PAD and by the induction of reduced blood flow. Multiple components of the locomotor system must cooperate to produce gait. Variability in gait provides insight into the ability of patients with PAD-IC to flexibly adapt to continuously changing movement environmental conditions. Gait variability is commonly used as an indicator of overall health of the biological system in older and pathological populations [23, 77, 78]. Previous studies found that patients with PAD-IC have increased amount and altered temporal structure of gait variability compared with healthy older controls [7, 8]. Gait alterations in patients with PAD-IC are consistently present prior to the onset of claudication pain and even from the initial steps of the patient when the oxygen levels in the legs have not been challenged yet [6–8, 44]. Our current study is the first to attempt to dissect the potential contribution of reduced blood flow which many investigators feel is a key mechanism operating to produce the gait alterations of PAD. Results of the comparisons between patients with PAD-IC and healthy older control groups are in agreement with previous studies from our laboratory, with patients with PAD-IC demonstrating increased amount and altered temporal structure of variability as compared with healthy older controls [7, 8]. The actual values of gait variability parameters of current study are also similar as previously reported gait variability parameters [7, 8].

The existence of group differences across conditions, as indicated by several significant interactions, suggests that factors other than reduced blood flow contribute to gait variability differences seen in patients with PAD-IC. Gait variability in the reduced blood flow condition of healthy older controls, which mimics the arterial blood flow supply-demand imbalance seen in patients with PAD-IC, was significantly different than that of the PAD-IC reduced blood flow condition. Our first study investigating the effect of induced vascular occlusion on gait variability in healthy young forecast such a finding [46]. Our previous studies compared the percentage change from pain free to reduced blood flow in healthy young [46] and the percentage difference from pain free to reduced blood flow between the healthy young and patients with PAD-IC [47]. While the reduced blood flow condition was significantly different than the pain free condition in healthy young, the patients with PAD-IC had significantly larger percentage differences in gait variability as compared to the increases seen in the young [46, 47]. For example, the percentage increases averaged across the ankle, knee, and hip for the healthy young were 21% for the largest Lyapunov exponent, 26% for the standard deviation, and 22% for the coefficient of variation. The same percent differences from healthy young to the PAD group in the pain free condition were 48% for the largest Lyapunov exponent, 62% for the standard deviation, and 99% for the coefficient of variation. This result is further supported in a similar comparison of gait variability changes from pain free to reduced blood flow in healthy older adults. The significantly larger percentage differences in gait variability, as compared with the increases seen in the young during reduced blood flow, suggest that reduced blood flow is only one mechanism contributing to differences between patients with PAD-IC and healthy older controls. The results of the direct statistical comparison between patients with PAD-IC and healthy older controls during the pain free and reduced blood flow walking conditions in the current study confirms and further advance the observations of the previous study. Accordingly, gait variability alterations seen in patients with PAD-IC must be further influenced by other disease-related mechanisms, in addition to being impacted by reduced blood flow.

Maintaining an optimal “state” of gait variability is desirable because such a state represents a healthy neuromusculoskeletal system that adapts to perturbations that occur during typical movement situations. Alterations in gait variability happen because of changes in the locomotor system. This includes changes to the movement generation and control pathways of the muscular and nervous systems and the circulatory system that supplies them. There is extensive evidence of damage to muscular and nerves system in the lower extremities of PAD patients, so it is likely that gait variability is also affected by these changes [36, 40, 41, 79]. In the muscular system of patients with PAD, skeletal muscles exhibit atrophy, denervation, and defective mitochondrial bioenergetics [40–43, 45, 79–82]. Changes to the lower extremity nervous system in patients with PAD include axonal nerve loss as indicated by electrodiagnostic and muscle strength and control abnormalities [16, 83]. How the muscular and nervous systems of patients with PAD change as the disease progresses or following treatment to restore blood flow are the obvious next steps of investigation.

An additional observation of importance includes the findings at the ankle joint time series. The current study demonstrated only one significant main effect for the ankle. Specifically, the patients with PAD-IC group have significantly increased values of the largest Lyapunov exponent as compared with healthy older controls. Several gait studies reported that the ankle joint is significantly altered in patients with PAD-IC when compared to healthy older controls [11, 12, 47, 84]. In the current study, alterations at the ankle are apparent through multiple significant interactions. Results of the post hoc analysis revealed decreased values for the coefficient of variation of ankle range of motion and the approximate entropy of the ankle joint time series for the PAD-IC reduced blood flow condition as compared with the PAD-IC pain free and healthy older controls reduced blood flow conditions. Therefore, the healthy older controls were able to maintain the level of variability at the ankle during the reduced blood flow condition, while patients with PAD-IC had reduced variability. Investigation of other measures of both amount and temporal structure of variability reveals that in patients with PAD-IC, all ankle variables decreased from the pain free to the reduced blood flow condition, although not significantly. These trends of decreased values for measures of amount and temporal structure of variability of the ankle joint time series are in agreement with our previous study investigating the effect of claudication pain [47]. In that study, we suggested that patients with PAD may attempt to decrease the use of the calf muscles, the typical location for experiencing claudication pain. It is also possible that patients with PAD are so limited during claudication pain that even the abnormal movement patients exhibited during pain free walking is not possible to maintain. Previous study also shows that electromyography amplitude of medial gastrocnemius and tibialis anterior significantly increased during the painful walking compared to pain-free walking in patients with intermittent claudicating [85]. Future studies could assess the changes in calf and other lower extremity muscles activity before and after the onset of claudication pain to investigate how their functions alter due to the claudication pain.

Results of the receiver operating characteristics curve analysis demonstrate that multiple gait variability parameters have potential for predicting PAD disease status (presence or absence of PAD). Interestingly, three of the four dependent variables assessing variability of hip joint movement were significantly related with disease status. The hip variability was the strongest predictive value compared to six significant gait variability parameters. The largest Lyapunov exponent for the ankle, knee, and the hip joint time series were significantly related with disease status. This suggests that this nonlinear measure is more strongly associated with disease status, regardless of the joint being observed.

Receiver operating characteristics curve analysis has been used extensively to predict lower extremity injuries, including falling in the older adults [73, 86–88]. In comparison with previous studies using functional tests to identify individuals at risk for falls, the current study has equal or better values of sensitivity and specificity. For example, Thrane et al. examined the predictive value of the timed up and go test for determining fall risk [86]. Sensitivity values in this study ranged from 11% to 44% and specificity varied from 58% to 98%. Sensitivity in our study went from 47% to 90% and specificity ranged from 63% to 97%. Therefore, our results demonstrate that gait variability is a sensitive and specific assessment of the lower extremity mobility problems experienced by patients with PAD-IC. Although several variables demonstrated a significant relationship with disease status, future studies should explore multivariate analysis and machine learning models, as the combination of multiple gait variability parameters might yield even better diagnostic capabilities.

The difficulty in interpreting the results of our study lies in the classifications of our subject conditions. Our study sought to determine the effect of reduced blood flow on gait variability alterations that occur in patients with PAD-IC. Therefore, we examined both patients with PAD-IC and healthy older controls during pain free and reduced blood flow conditions. Both groups had lower extremity blood flow assessed at rest and we acknowledge that patients with PAD-IC have abnormal blood flow even at rest. Similarly, we acknowledge that the vascular occlusion procedure performed to reduce lower extremity blood flow in the healthy older controls did not lead to hemodynamics identical to those experienced by patients with PAD-IC. However, the approach was effective in producing perturbed blood flow and significant alterations in gait during the post occlusion condition. Furthermore, the reductions in blood flow delivery from a rested state to an exerted state, documented by the ankle-brachial indices during rest and exercise in patients with PAD [89, 90], are reflective of the lower extremity hemodynamics during the PAD-IC pain free and PAD-IC reduced blood flow conditions.

Although it was not possible to continuously monitor the ankle-brachial index of healthy older controls during the reduced blood flow condition, the current protocol created a critical episode of clinical ischemia consistent with reduced arterial inflow. The vascular occlusion procedure was modeled after the vascular laboratory technique for inducing ischemia and reperfusion, which creates a supply-demand imbalance similar to that experienced by patients with PAD [56–61]. Therefore, our study provides important insights into the effect of reduced blood flow on gait variability parameters, regardless of the difficulties in simulating the blood flow status of PAD in healthy older controls. The vascular occlusion method used in our study could be validated by newly available technologies, such as a tissue oximeter, to better document blood flow delivery to the calf muscles during pain free and reduced blood flow conditions.

## 5. Conclusions

The results of our study overall demonstrate that gait variability is affected both by PAD and by the induction of an episode of reduced blood flow. The presence of group differences across conditions supports the findings of our previous studies that a number of other factors, in addition to reduced blood flow, contribute to gait variability differences between healthy older controls and those with PAD-IC. These gait variability changes in healthy older controls caused by the induced vascular occlusion should highlight the importance of screening older individuals at risk of developing PAD, as potential mobility problems and altered gait variability can develop with reductions of blood flow, even if no claudication symptoms exist. Gait variability parameters have potential diagnostic value, as some gait variability measures had 90.0% probability of identifying patients with PAD-IC. Future research should investigate this further, including incorporating multivariate prediction models to more accurately classify patients with PAD-IC and healthy older individuals.
